# A diagnostic signatures for intervertebral disc degeneration using TNFAIP6 and COL6A2 based on single-cell RNA-seq and bulk RNA-seq analyses

**DOI:** 10.1080/07853890.2024.2443568

**Published:** 2024-12-20

**Authors:** Hong-Mei Song, Zuo-Wei Li, Qin Huang, Chun-Gen Wu, Ming-Hua Li, Jun-Kang Shen

**Affiliations:** a Department of Radiology, The Second Affiliated Hospital of Soochow University, Suzhou, China; b Department of Interventional Radiology, Shanghai Sixth People’s Hospital Affiliated to Shanghai Jiao Tong University School of Medicine, Shanghai, China; c Department of Urological Surgery, Shanghai Sixth People’s Hospital Affiliated to Shanghai Jiao Tong University School of Medicine, Shanghai, China; d Department of Pathology, Shanghai Sixth People’s Hospital Affiliated to Shanghai Jiao Tong University School of Medicine, Shanghai, China

**Keywords:** Intervertebral disc degeneration (IVDD), differentially expressed genes (DEGs), hub genes, diagnostic model, signaling pathway

## Abstract

**Objectives:**

Intervertebral disc degeneration (IVDD) is a prevalent degenerative condition associated with a high incidence rate of low back pain and disability. This study aimed to identify potential biomarkers and signaling pathways associated with IVDD.

**Methods:**

Biomarkers were discerned through bulk-RNA and single-cell RNA sequencing (scRNA-Seq) investigations of IVDD cases from the Gene Expression Omnibus (GEO) database. Following this, two central genes were identified. Furthermore, gene set enrichment analysis (GSEA) and receiver operating characteristic (ROC) curve analysis were conducted. The transcriptional factor (TF) derived from nucleus pulposus (NP) cells was examined through the DoRothEA R package. RT-qPCR and IHC techniques were employed to confirm the expression of the two hub genes and their associated genes in tissue samples.

**Results:**

The proteins Tumor necrosis factor-inducible gene 6 protein (TNFAIP6) and collagen VI-α2 (COL6A2) were frequently analyzed using a combination of DEGs from datasets GSE70362, GSE124272, and scRNA-seq. Examination of gene expression across multiple datasets indicated significant differences in TNFAIP6 and COL6A2 levels in IVDD compared to control or normal groups (*p* < 0.05). These two central genes demonstrated strong diagnostic utility in the training cohort and reliable predictive value in the validation datasets. Our study verified the potential role of ZEB2 as a TF in regulating two key genes associated with IVDD. Furthermore, qPCR and IHC confirmed elevated expression levels of the hub genes and transcription factor.

**Conclusion:**

We identified biomarkers, specifically TNFAIP6 and COL6A2, that have the potential to predict disease activity and aid in the diagnosis of IVDD.

## 1. Introduction

Intervertebral disc degeneration (IVDD) is a progressive and chronic condition that is associated with complications such as herniation, spinal canal stenosis, and degenerative spondylolisthesis [1–4]. IVDD is a significant contributor to low back pain, characterized by structural damage, apoptosis of nucleus pulposus (NP) cells, and degradation of the extracellular matrix (ECM) [5]. Despite current treatment options including conservative therapies and surgical interventions, outcomes are often suboptimal and can be complicated by various factors [6,7]. Numerous studies have explored the pathological alterations, molecular pathways, and biomarkers associated with intervertebral disc degeneration (IVDD) [8–14]; nevertheless, these investigations and biomarkers remain insufficient for practical application in clinical settings. The identification of novel signatures is imperative for the effective monitoring of IVDD progression.

IVDD is a complex condition that presents challenges in clinical evaluation due to limited availability of clinical samples. Biomarker panels, such as SOX9, FLVCR1, NR5A1, UCHL1 [5], CHI3L1, KRT19, collagen VI-α2 (COL6A2), DPT, tumor necrosis factor-inducible gene 6 protein (TNFAIP6), COL11A2 [9], CYP27A1, FAR2, CYP1B1 [11], ID1, PTPRK, RAP2C [13], FPR1, RLN1, S100Z, IFNGR2, KLRK1, CTSS [15], interleukin-1β (IL-1β), LYN, and NAMPT [16], have been used as diagnostic prediction markers for IVDD. Similarly, studies on IVDD pathology mainly focused on immune infiltration [13,16–18], tissue fibrosis [19], inflammatory responses [15,16,20], oxidative stress [21], the anabolism of ECM [22], insufficient transport of metabolites [23], enhanced aggrecan and collagen degradation [24], and the structural and functional abnormalities in mitochondria [25]. Biological markers and therapeutic interventions aimed at modulating inflammation have garnered increased interest in the scientific community. Research focusing on biomarkers related to nucleus pulposus and the inflammatory response has provided valuable insights into the diagnosis and treatment of IVDD.

Presently, researchers are employing scRNA-Seq analysis of IVDD tissue as a promising approach to uncovering the underlying mechanisms of IVDD [26–30]. Due to the small number of cases and differences in sampling, these results varied considerably.

**Figure 1.**
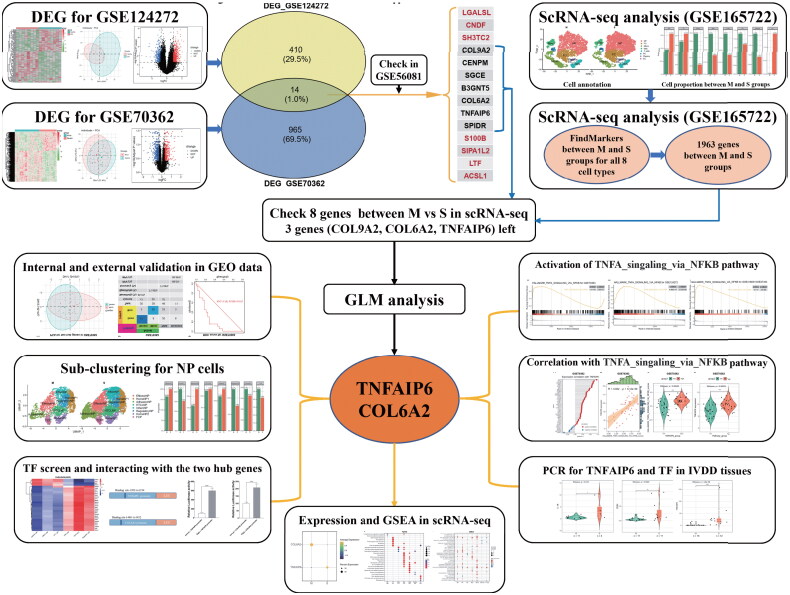
Workflow of the study. The study integrates human scRNA-seq and GEO bulk-RNA data to reconstruct the IVDD-specific work. A generalized linear model (regression) model was employed to prioritize the hub gene in the work. The potential function and mechanisms of identified genes were examined through GEO data validation, transcriptional factor (TF) and GSEA analysis, as well as whole blood and tissue tests.

Hence, we integrated bulk RNA-seq and scRNA-seq analyses of IVDD-related tissues and whole blood samples to elucidate key hub genes implicated in the pathogenesis of IVDD. Subsequent comprehensive functional and enrichment analyses of these hub genes were conducted (Figure 1). The reliability of these hub genes was further confirmed through validation using both GEO datasets and our own cohort. Additionally, transcription factors (TF) associated with the identified hub genes and TNFAIP6-related signaling pathways were assessed for their role in the progression of IVDD. These findings have the potential to inform the identification of biomarkers and therapeutic targets for IVDD.

## 2. Materials and methods

### 2.1. Data availability

The microarray datasets related to IVDD were reviewed and acquired from the gene expression omnibus (GEO, https://www.ncbi.nlm.nih.gov/geo/), a publicly available genomics database that encompasses high-throughput sequencing data, chips, and microarray data. The IVDD-associated datasets examined in this research comprised one single-cell RNA sequencing dataset (GSE165722) and six bulk RNA datasets (GSE70362, GSE124272, GSE56081, GSE34095, GSE41883, and GSE27494). For GSE165722, patients were characterized into two groups according to Pfirrmann Grading (Minor (M) group: II-III and Severe (S) group: IV-V). Considering the small number of samples in bulk RNA-sequencing data, we removed the batch effect by merged GSE56081 and GSE34095 and GSE41883 and GSE27494 *via* the ‘limma’ package.

Dataset GSE70362 (GPL17810 platform) included 28 minor and 20 severe IVDD tissue samples, dataset GSE56081 (GPL15314 platform) included five control samples and 5 IVDD samples, dataset GSE34095 (GPL96 platform) included three control samples and three IVDD samples, dataset GSE41883 (GPL1352 platform) included four disc cells samples and four TNF-a induced disc cells samples, dataset GSE27494 (GPL1352 platform) included four disc cells samples and four IL-1 induced disc cells samples, and dataset GSE124272 (GPL21185 platform) included 16 control and 16 IVDD-related peripheral whole blood samples (Table S1).

The datasets GSE70362, GSE124272, and GSE165722 (scRNA-Seq) were utilized for the selection of biomarkers. GSE70362 was employed as the training dataset, while GSE56081 + GSE34095 and GSE41883 + GSE27494 were utilized as validation datasets. GSE165722 was utilized as the single-cell dataset.

This study employed a publicly accessible dataset that had previously received ethical approval. Informed consent was secured from all participants, and the research was conducted in strict accordance with the principles delineated in the Declaration of Helsinki.

### 2.2. Differentially expressed genes analysis for bulk RNA-seq

The R package ‘Limma’ was utilized to identify differentially expressed genes (DEGs) between control and IVDD samples. Additionally, genes meeting the criteria of adjusted P-values or P-values < 0.05 and |log2FC| ≥ 1.5 were classified as statistically significant genes.

### 2.3. Single-cell sequencing analysis

The scRNA-seq data analysis was performed utilizing the software ‘Seurat’ (version 4.1.2). Cells deemed to be of low quality, as determined by criteria including minimum expression levels greater than 3, gene counts less than 200, and mitochondrial gene proportions exceeding 15%, were excluded from subsequent analysis. The remaining cells were employed for subsequent bioinformatic analysis, which included a principal component analysis (PCA) utilizing the top 2,000 variably expressed genes. Following this, the top 15 principal components were chosen for cell clustering analysis using the T-distributed stochastic neighbor embedding (t-SNE) method. Marker genes were identified based on a |log2 (fold change)| exceeding 1 and an adjusted p-value below 0.05 [31].

### 2.4. Analysis of cell clustering and subclustering

The FindClusters function within the Seurat software package was utilized to identify cell clusters and subclusters at appropriate resolutions. The Seurat Function FindAllMarkers, incorporated in version 4.1.2, was employed to conduct differential expression analysis on the complete cell cluster in order to extract relevant information pertaining to IVDD status. The cell clusters and subclusters were annotated with highly-expressed genes, marker genes identified through differential expression gene (DEG) analysis, and reported cellular markers [31]. Furthermore, the DEG analysis was applied to all cell clusters utilizing the FindMarkers function integrated within Seurat (version 4.1.2) to discern valuable information indicative of the IVDD state.

### 2.5. Functional enrichment analysis

We performed a Gene Ontology (GO) and Kyoto Encyclopedia of Genes and Genomes (KEGG) analysis on the differential marker genes identified between subclusters utilizing ClusterProfiler 4.0 in the R programming language [32]. The COSG package [33] was employed to analyze the differential marker genes across subclusters. The gene sets utilized in this analysis were sourced from the Molecular Signatures Database (MSigDB) (https://www.gseamsigdb.org/ gsea/downloads.jsp) [34]. We used the Scillus package to perform Gene Set Enrichment Analysis (GSEA) on the differential marker gene expression among subclusters (https://github.com/xmc811/ Scillus). The TF in NP was analyzed using the DoRothEA R package (https://github.com/saezlab/dorothea) [35], which is a gene regulatory network (GRN) containing signed transcription factor (TF) - target gene interactions.

### 2.6. Selection of hub genes, model construction and nomogram establishment

Logistic least absolute shrinkage and selection operator (LASSO) regression was utilized for the identification of hub genes in this study. The modeling process involved the use of a generalized linear model (regression). The diagnostic performance of the model was assessed through the analysis of receiver operating characteristic (ROC) curves and calculation of the area under the curve (AUC) using a generalized linear model. A nomogram was constructed with the characteristic genes using the rms package, and its accuracy was evaluated against a calibration curve. Furthermore, the clinical utility of the nomogram was assessed through a decision curve analysis.

### 2.7. Quantitative real-time PCR (RT-qPCR)

A total of 36 whole blood samples (13 normal and 23 IVDD samples) and 30 IVDD tissues (18 minor and 12 severe IVDD samples) with MRI-confirmed disc herniation were collected from the Shanghai Sixth People’s Hospital Affiliated to Shanghai Jiao Tong University School of Medicine. The Ethics Committee of our hospital approved this study (Approve No: 2023-KY-167(K)). Moreover, the respective patient provided written informed consent.qPCR for IVDD tissues: Homogenize tissue samples in 1 mL of RNA Extraction per 20 mg of tissue using Tissue homogenizer. The sample volume should not exceed 10% of the volume of RNA Extraction used for the homogenization. The total RNA of cells was isolated using TRIzol reagent (Invitrogen, USA) according to the manufacturer’s manual. Next, cDNA was reverse transcribed from RNA using PrimeScript RT Master Mix (Takara). The qPCR was performed with an SYBR Green PCR kit (Takara). The primer sequences for IVDD tissues were as follows (5′-3′): GAPDH (forward: 5′- GGAAGCTTGTCATCAATGGAAATC-3′, reverse: 5′-TGATGACCCTTTT- GGCTCCC-3′), TNFAIP6 (forward: 5′- CTTGAAGATGACCCAGGTTGCT-3′, reverse: 5′- CTCATCT- CCACAGTATCTTCCCAC- 3′), IL-1B (forward: 5′- CGATCACTGAACTGC- ACGCTC-3′, reverse: 5′- ACAAAGGACATGGAGAACACCACTT-3′), ZEB2 (forward: 5′- AGGAA- GATGAAATAAGGGAGGGT-3′, reverse: 5′-TCACTGTACCATTGTTAATTG- CGG-3′) synthesized by shanghai Generay Biotech. The GAPDH gene was used as an internal reference, and the target gene expression was determined with the 2^-ΔΔ^Ct method [36].

### 2.8. Immunohistochemistry (IHC) analysis

The tissues were fixed using a 4% paraformaldehyde solution for a duration of 15 min, followed by immersion in paraffin and subsequent cutting into slices with an average thickness of 4 μm. After dewaxing and dehydration, the antigens were extracted. The resulting slices were then treated with a 3% hydrogen peroxide solution for 20 min and blocked at room temperature for 15 min using a 5% BSA solution. Subsequently, the slices were incubated overnight at 4 °C with anti-IL1β (26048-1-AP; 1:100; Proteintech), anti-ZEB2 (14026-1-AP; 1:100; Proteintech), anti-TNFAIP6 (PA5-75332; 1:100; ThermoFisher), and anti-COL6A2 (14853-1-AP; 1:50; Proteintech) antibodies, diluted in Antibody Diluent Solution (Life-iLab, Shanghai, China). The segments were subjected to color development using a color-developing agent for a duration of 3-15 min. Subsequently, they were washed, redyed, dehydrated, rendered transparent, and sealed in a sequential manner. These processed segments were then examined using SP kits (Solarbio, Beijing, China). Finally, the slices were observed and photographed under a light microscope. The staining results were analyzed using Image-J software (NIH, United States). Semiquantitative analyses were conducted by calculating the average optical density (AOD) of positive staining for TNFAIP6, COL6A2, IL1B, and ZEB2. The AOD was determined by comparing the integral optical density (IOD) with the corresponding area [37].

### 2.9. Statistical analysis

Bioinformatics analyses and R packages were performed using R software version 4.2.0. Student’s t-test was utilized to compare the means of normally distributed variables between two groups, while the Wilcoxon test was employed for comparing non-normally distributed data. Significance levels were denoted as *p < 0.05, **p < 0.01, and ***p < 0.001.

## 3. Results

### 3.1. Identification of DEGs of GEO datasets

**Figure 2.**
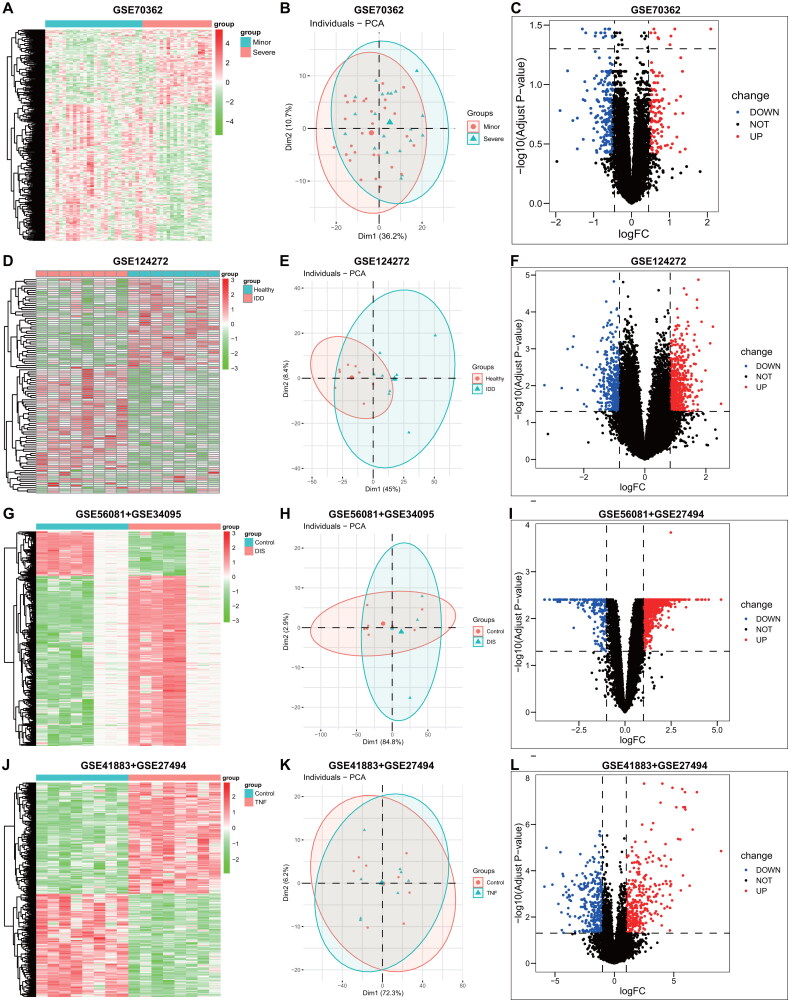
Heatmap, PCA and volcano plots of the IVDD with GEO datasets between control and IVDD samples. (**A-C**) Heatmap, PCA and volcano plots from GSE70362. (**D-F**) Heatmap, PCA and volcano plots from GSE124272. (**G-I**) Heatmap, PCA and volcano plots from GSE56081 + GSE34095. (**J-L**) Heatmap, PCA and volcano plots from GSE41883 + GSE27494.

The study procedure flow chart was presented in Figure 1, with differential expression gene (DEG) analyses performed on the GEO datasets GSE70362, GSE124272, GSE56081 + GSE34095, and GSE41883 + GSE27494, resulting in 979, 424, 927, and 619 DEGs, respectively. Figure 2 display the heatmap, PCA, and volcano plots for these datasets.

### 3.2. Profiling of scRNA-seq

ScRNA-seq data sourced from GEO165722 was utilized to analyze NP tissues from eight IVDD patients exhibiting varying degrees of degeneration. The patients were categorized into two groups, M (Pfirrmann Grading II-III) and S (Pfirrmann Grading IV-V). A total of 36,327 cell samples were collected, with 19,600 cells in the M group and 20,327 cells in the S group (Table S2). Following quality control measures, 17,858 cell samples were retained, consisting of 7,758 cells in the M group and 10,100 cells in the S group (Table S3), for subsequent analysis.

**Figure 3.**
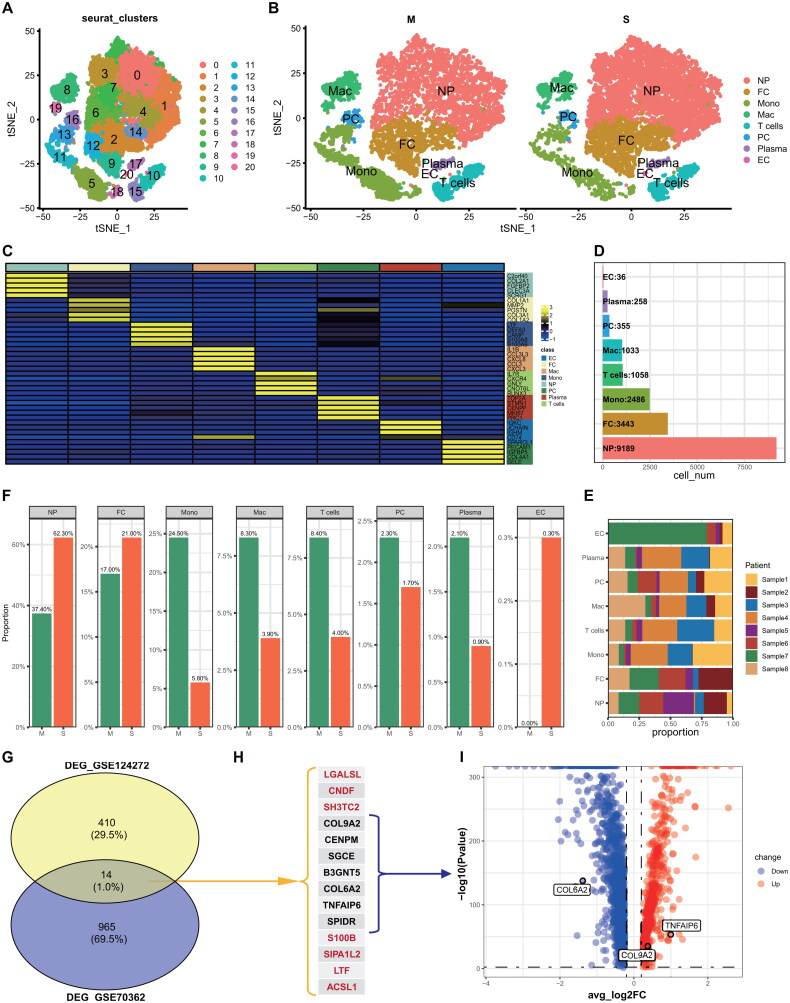
scRNA-Seq analysis and biomarker selection. (**A**) t-SNE plot colored by 21 cell types. **(B)** the t-SNE plot of IVDD-M and IVDD-S groups colored by eight cell clusters. **(C)** The heatmap shows the top five markers of the eight clusters. **(D & E)** The boxplot shows the cell numbers and proportion of the eight cell clusters. **(F)** The boxplot displays the cell proportion between the M and S groups of the eight cell clusters. **(G & H)** The venn plot exhibits 14 DEGs from the intersection of the two GEO datasets (GSE70362 and GSE124272). **(I)** The volcano plot of scRNA-seq analysis between IVDD-M and IVDD-S groups showed three significantly different DEGs. **Note:** IVDD = intervertebral disc degeneration; M = Minor; and S = Severe.

After addressing batch effects and normalizing the data using the Harmony R package, a total of 21 distinct cell clusters with unique gene expression profiles were identified at a resolution of 1.0 (Figure 3 A). Subsequently, these clusters were categorized into eight cell clusters, including NP cells, fibroblast cells (FC), endothelial cells (EC), macrophage (Mac), monocytes (Mono), proliferating cells (PC), T cells, and plasma cells (Figure 3 B-E). The marker genes on comparison all of the 7 cell clusters in the single-cell data can be found in Table S4. NP cells were found in multiple subpopulations with a high number of cells, while endothelial cells (EC) were present in fewer subpopulations with lower cell numbers (Figure 3A and D). These findings suggest that NP cells may play a significant role in IVDD.

Upon comparing the cellular distinctions between the M and S groups, it was observed that the expression levels of NP, FC, and EC were elevated, while the remaining five cell clusters exhibited decreased expression in the S group (Figure 3 F). The ‘FindAllMarkers’ function integrated within Seurat was utilized to identify differentially expressed genes (DEGs) across all cell clusters. Subsequently, the FindMarkers function was employed to identify DEGs between the M and S groups, resulting in the identification of 1963 DEGs (Table S5).

### 3.3. Biomarkers selection from GEO and scRNA-seq

Through scRNA-seq analysis, we validated the enrichment of nucleus pulposus (NP) cells and their significant involvement in IVDD patients. Subsequently, we endeavored to ascertain robust biomarkers for the diagnosis and prognostication of IVDD in comparison to control subjects by constructing a predictive model utilizing ROC analysis. Integration of DEGs identified through scRNA-seq with data from two GEO datasets (GSE70362 and GSE124272) enabled the development of a comprehensive model for accurate NP diagnosis and prediction.

Initially, 1963 DEGs between M and S were identified as the primary feature input from single-cell RNA sequencing analysis (Table S5). Subsequently, a total of 14 common shared DEGs (LGALSL, CNDF, SH3TC2, COL9A2, CENPM, SGCE, B3GNT5, COL6A2, TNFAIP6, SPIDR, S100B, SIPA1L2, LTF, and ACSL1) were identified through the intersection of the DEGs and two GEO datasets (Figure 3 G). Upon further examination of these DEGs in GSE56081, it was observed that only seven DEGs (COL9A2, CENPM, SGCE, B3GNT5, COL6A2, TNFAIP6, SPIDR) were expressed in this particular GEO dataset (Figure 3 H). Next, seven DEGs were identified from a pool of 1963 DEGs through scRNA-seq, leading to the selection of three specific DEGs (COL9A2, COL6A2, and TNFAIP6) for further investigation (Figure 3 I). Subsequent efforts involved exploring various combinations to determine the optimal area under the curve (AUC) value using these three signatures in both training datasets (GSE70362 and GSE124272) and validation datasets (GSE56081 + GSE34095 and GSE41883 + GSE27494) from the GEO. Ultimately, COL6A2 and TNFAIP6 emerged as the most effective signatures.

### 3.4. The expression and signaling pathways of the two genes in scRNA-seq

**Figure 4.**
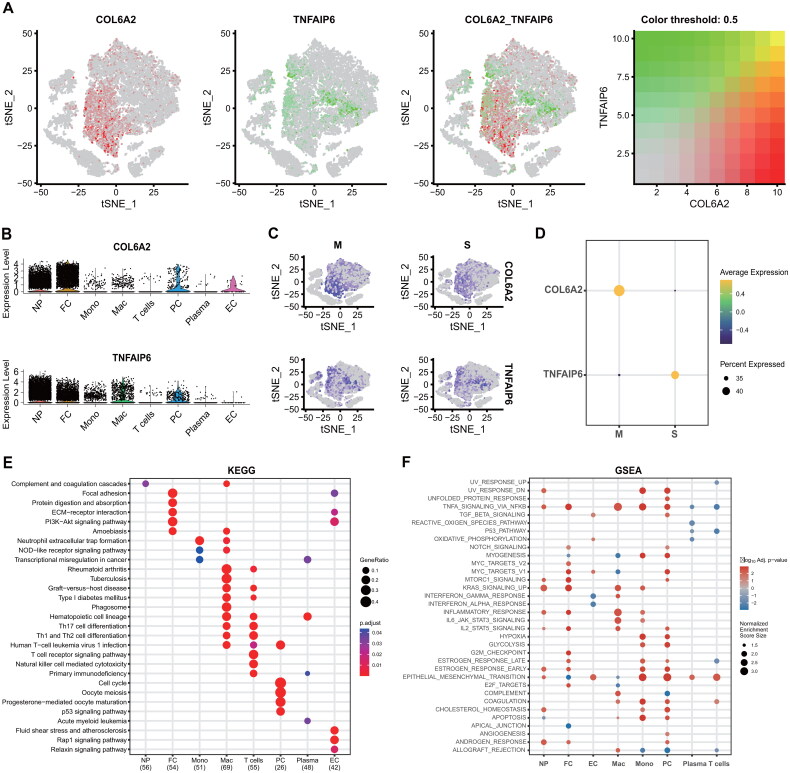
The expression and signaling pathways in scRNA-seq. (**A-B**) The plots display the expression of COL6A2 and TNFAIP6 in scRNA-seq. (C-**D**) Compared with the IVDD-M group, the COL6A2 expression was downregulated, and the TNFAIP6 expression was upregulated in the IVDD-S group. (**E**) The KEGG plot shows the KEGG pathway in all eight cell clusters. (**F**) The GSEA displays the signaling pathways in all eight cell clusters. **Note:** IVDD = Intervertebral disc degeneration; M = Minor; and S = Severe.

Following the identification of the two signatures, we conducted an initial investigation into their expression in scRNA-seq. As illustrated in Figure 4 A-B, the genes COL6A2 and TNFAIP6 exhibited predominant expression in NA and FC cell populations. In comparison to the IVDD-M group, the expression of COL6A2 was downregulated, while TNFAIP6 expression was upregulated in the IVDD-S group (Figure 4 C-D). Subsequently, KEGG analysis was performed on all identified cell clusters. Nucleus pulposus (NP) cells demonstrated enrichment in complement and coagulation cascades, whereas the FC displayed enrichment in focal adhesion, protein digestion and absorption, ECM-receptor interaction, PI3K-Akt signaling pathway, and amoebiasis (Figure 4E). Lastly, GSEA was executed for all the eight clusters from all DEGs between M and S groups. The GSEA showed that the NP and FC commonly shared TNFA_singaling_*via*_NFKB, MTORC1_singnaling, KRAS_signaling_up, inflammatory_response, IL2_STAT5_signaling, estrogen_response_early, epithelial_mesenchymal_transtion and androgne_response (Figure 4F). These results indicated the involvement of the multiplex pathways and signals in the pathophysiology of IVDD progression.

### 3.5. Diagnostic model construction and training for IVDD

The cohort GEO70362 was utilized as the training dataset, while the cohort GEO124272 was utilized as the internal validation dataset for the purpose of developing and validating signatures to diagnose and predict the progression of IVDD. Subsequently, a diagnostic prediction model was constructed based on a generalized linear model (regression) incorporating COL6A2 and TNFAIP6 signatures. The model score was −3.837+ (-0.944 * COL6A2 + 1.072 * TNFAIP6) (Table S6).

**Figure 5.**
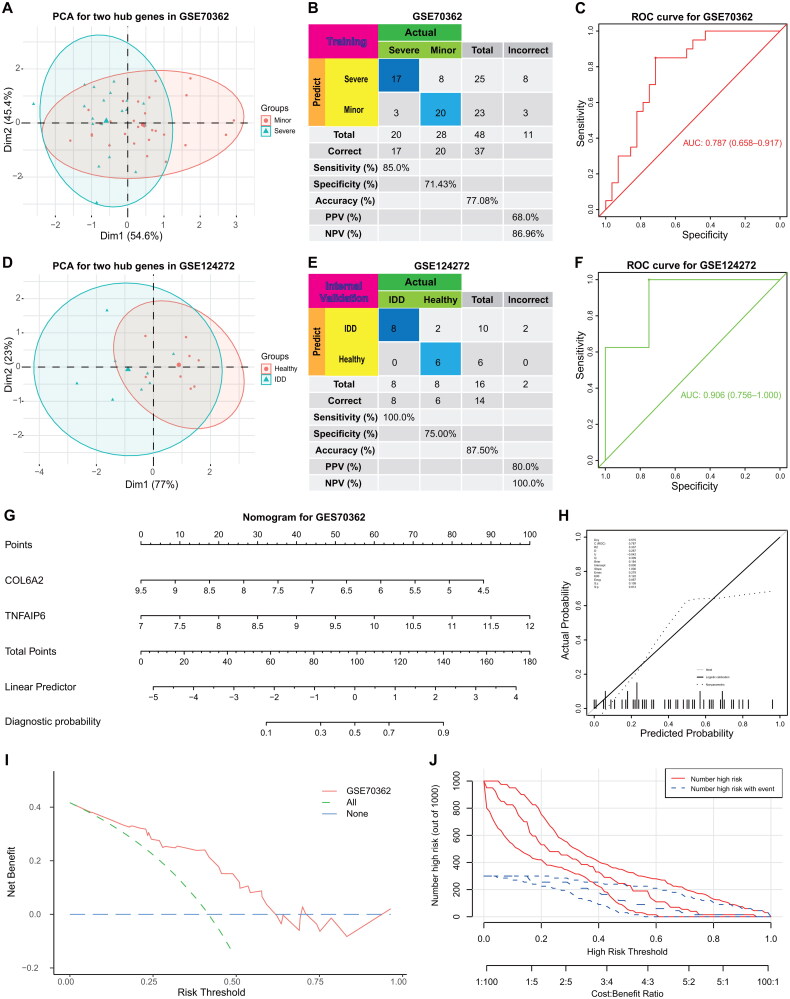
Diagnostic model for IVDD. (**A**) The expression of the COL6A2 and TNFAIP6 between IVDD-M and IVDD-S in the GEO70362 training cohort. (**B**) The confusion matrix for the GSE70362 training cohort. (**C**) Assessment of the diagnostic predictive accuracy of the training cohort for the two signatures using ROC curves (IVDD-M = 28, IVDD-S = 20, and AUC = 0.787). (**D**) The expression of the COL6A2 and TNFAIP6 between IVDD-related peripheral whole blood samples and control samples in the GEO124272 internal validation cohort. (**E**) The confusion matrix for the GSE124272 training cohort. (F) Assessment of the diagnostic predictive accuracy for the internal validation cohort for the two signatures using ROC curves (IVDD = 8, control = 8, and AUC = 0.906). (**G**) A constructed nomogram for diagnostic prediction of IVDD. (**H**) The calibration curve, **(I)** DCA curve and **(J)** clinical impact curve for assessing the nomogram’s performance.

Initially, we examined the differential expression of two biomarkers in IVDD-M and IVDD-S samples within the GEO70362 dataset. Subsequent analysis utilizing PCA (Figure 5A), confusion matrix (Figure 5B), and ROC plots (Figure 5C) demonstrated the discriminatory potential of the two biomarkers in distinguishing between the two groups. Additionally, we assessed the utility of these biomarkers in IVDD-related peripheral whole blood samples from the GSE124272 dataset, revealing their ability to effectively differentiate IVDD samples from control samples (Figure 5 D-F).

The findings indicated that the two hub genes exhibited favorable diagnostic efficacy. Furthermore, a nomogram incorporating these key signatures was developed for the diagnosis of IVDD (Figure 5G). Subsequent evaluation of the nomogram’s accuracy through calibration curve analysis demonstrated close alignment between the bias-corrected curve and the ideal curve (Figure 5H). Additionally, assessments using decision curve analysis and clinical impact curve indicated the robust performance of the diagnostic model (Figure 5 1-J). Notably, the model demonstrated superior net benefit rates at thresholds exceeding 0.50, as depicted in the accompanying figure (Figure 5J).

### 3.6. External cohorts for prediction of IVDD

**Figure 6.**
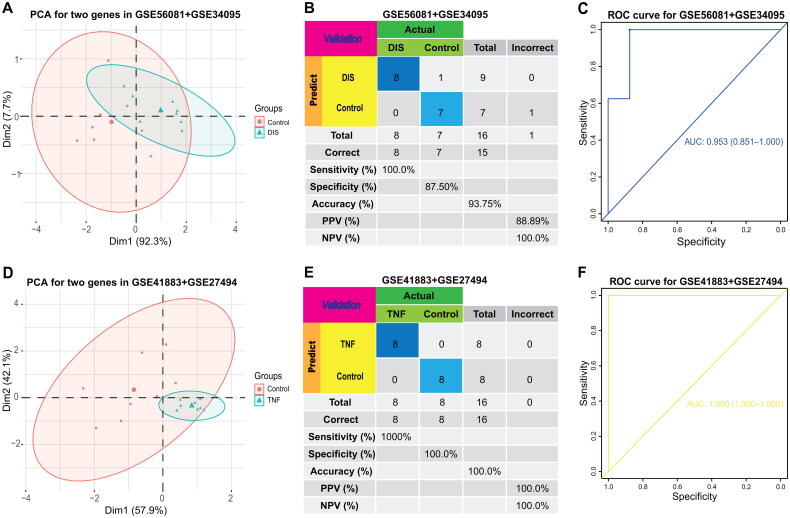
External diagnostic validation for IVDD. (**A**) The expression of the COL6A2 and TNFAIP6 between IVDD and normal tissues in the GSE56081+ GSE34095 external validation cohort. (**B**) The confusion matrix for GSE56081+ GSE34095 external validation cohort. (**C**) Assessment of the diagnostic predictive accuracy for the training cohort for the two signatures using ROC curves (IVDD = 8, Healthy = 8, and AUC = 0.953). (**D**) The expression of the COL6A2 and TNFAIP6 between treated disc cells and control disc cells in the GSE41883 + GSE27494 external validation cohort. (**E**) The confusion matrix for GSE41883 + GSE27494 external validation cohort. (**F**) Assessment of the diagnostic predictive accuracy for the external validation cohort for the two signatures using ROC curves (Treated disc cells = 8, Control disc cell = 8, and AUC = 1).

Following this, we conducted an evaluation of the two signatures using two external GEO datasets (GSE56081+ GSE34095 and GSE41883 + GSE27494). The results depicted in Figure 6 A-C and Figure 6 D-F indicate that the two hub genes exhibited consistent high diagnostic accuracy in distinguishing IVDD samples from controls, with AUC values of 0.953 and 1.00 in the respective GEO datasets. These observations imply that the two hub genes have the potential to function as predictive biomarkers for IVDD.

### 3.7. NP cell subclustering and transcriptional factor (TF) analysis in scRNA-seq

**Figure 7.**
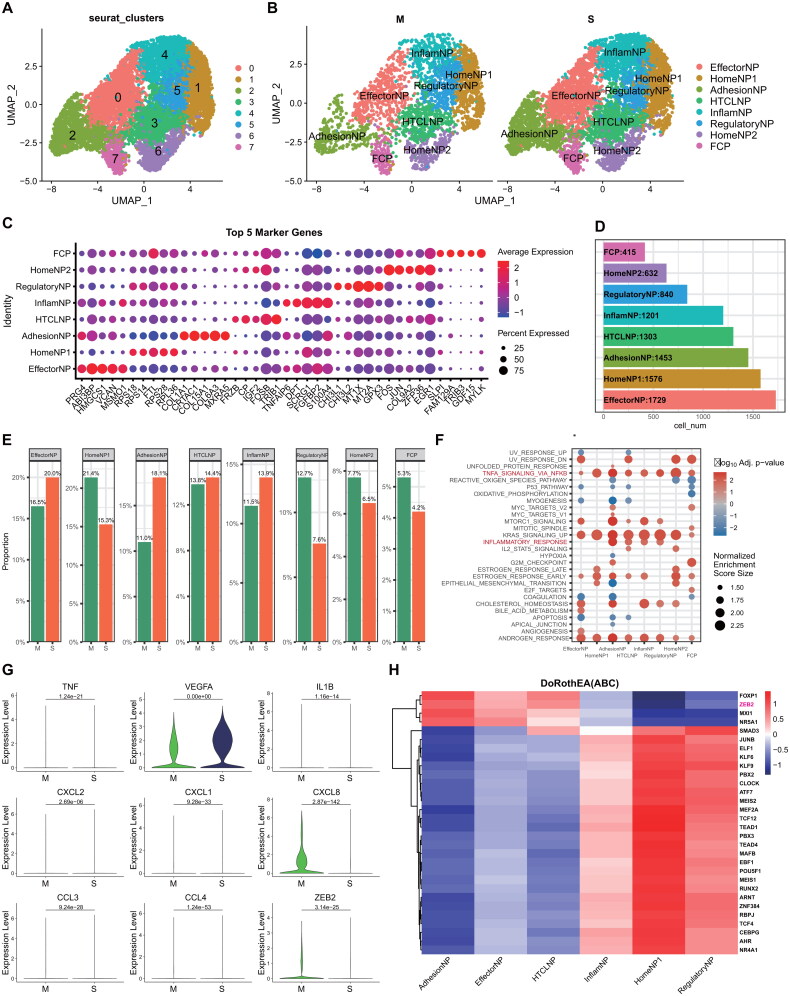
scRNA-Seq analysis for NP cell. **(A)** UMAP plot colored by seven cell types. **(B)** UMAP plot of M and S groups colored by seven cell clusters. **(C)** The heatmap shows the top five markers of the seven clusters. **(D)** The boxplot shows the cell numbers of the seven cell clusters. **(E)** The boxplot displays the cell proportion between the M and S groups of the seven cell clusters. **(F)** The GSEA of the seven NP cells clusters. **(G)** The violin plot exhibited the elevation of the pro-inflammatory cytokines in the S group in NP cells compared with the M group in the M group in NP cells. **(H)** The heatmap displayed the top 30 TF with the DoRothEA R package.

Nucleus pulposus (NP) cells constituted the predominant cell type in our study, accounting for 51.46% (9189/17858) of the total cells analyzed. The progression of intervertebral disc degeneration (IVDD) was found to be influenced by a complex network of multiplex pathways and signals. Subsequent sub-clustering and annotation of NP cells were conducted to elucidate the heterogeneity and phenotypic variations within the NP cell populations. We identified seven distinct NP clusters (EffectorNP, AdhesionNP, HTCLNP, InflamNP, RegulatoryNP, HomeNP1, HomeNP2, and FCP) with a resolution of 0.5 according to the literature [26–30] (Figure 7 A-D). The marker genes on comparison all of the 8 cell subclusters are shown in Table S7. In addition, compared to the M group, EffectorNP, AdhesionNP, HTCLNP, and InflamNP were elevated, whereas the other three subclusters were decreased in the S group (Figure 7E). According to the GSEA, TNFA_singaling_*via*_NFKB, KRAS_signaling_up, estrogen_response_early, and androgne_ response were activated in all the subclusters (Figure 7F). Next, an increase in pro-inflammatory cytokines, including TNF, VEGFA, IL1B, CCL3, CCL4, CXCL1, CXCL2, and CXCL8, was observed in the S group of NP cells (Figure 7G), suggesting a potential role for these cytokines in the progression of IVDD. To further elucidate the mechanisms underlying this increase in NP, we utilized the DoRothEA R package to analyze the active transcription factors (TF) in NP. The top 30 active TF were visualized as a heatmap (Figure 7H). By considering the cell proportions between the M and S groups (Figure 7E), it was determined that four TF (FOXP1, ZEB2, MXI1, and NR5A1) may partially account for the changes in cell proportion during NP development.

### 3.8. Exploring key TFs regulating the two hub genes

**Figure 8.**
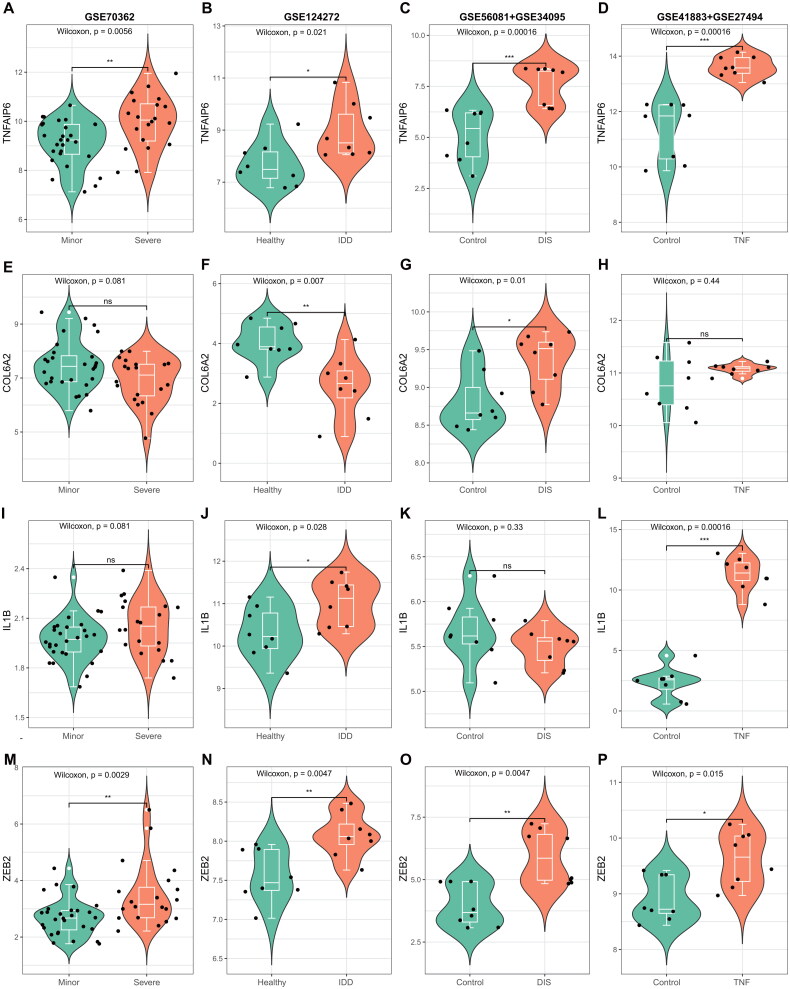
Examination of the levels of TNFAIP6, COL6A2, IL-1β, and ZEB2 expression across four GEO datasets. (**A-D**) Analysis the expression of TNFAIP6 in the following GEO datasets: GSE70362, GSE124272, GSE56081+ GSE34095, and GSE41883 + GSE27494. (**E-H**) Comparison of the expression of COL6A2 in the same four GEO datasets. (**I-L**) Assessment the expression of IL-1β in these datasets. (**M-P**) Analysis the expression of ZEB2 in the four GEO datasets.

The regulatory transcription factors (TFs) associated with the hub genes TNFAIP6 and COL6A2 were investigated by examining their expression levels, along with IL-1β and a key TF, in four GEO datasets (GSE70362, GSE124272, GSE56081+ GSE34095 and GSE41883 + GSE27494). The results depicted in Figure 8 indicate that TNFAIP6 exhibited higher expression levels in the IVDD-S group compared to the IVDD-M group across all four GEO datasets (Figure 8 A-D), while the expression of COL6A2 in the IVDD-S group varied among the datasets (Figure 8 E-H). Given the recognized importance of IL-1β as a key mediator in the process of intervertebral disc degeneration (IVDD) [16], the expression of IL-1β was subsequently evaluated in four Gene Expression Omnibus (GEO) datasets. As illustrated in Figure 8 1-L, the expression of IL-1β in the IVDD-S group was found to be upregulated in three of the four GEO datasets. Furthermore, based on the results obtained from the DoRothEA R package, we analyzed the mRNA expression levels of four transcription factors (TFs) in all four GEO datasets, revealing that only the expression levels of ZEB2 were consistently elevated across all case groups (Figure 8 M-P).

### 3.9. Verification of the interaction network between TF and the two hub genes

**Figure 9.**
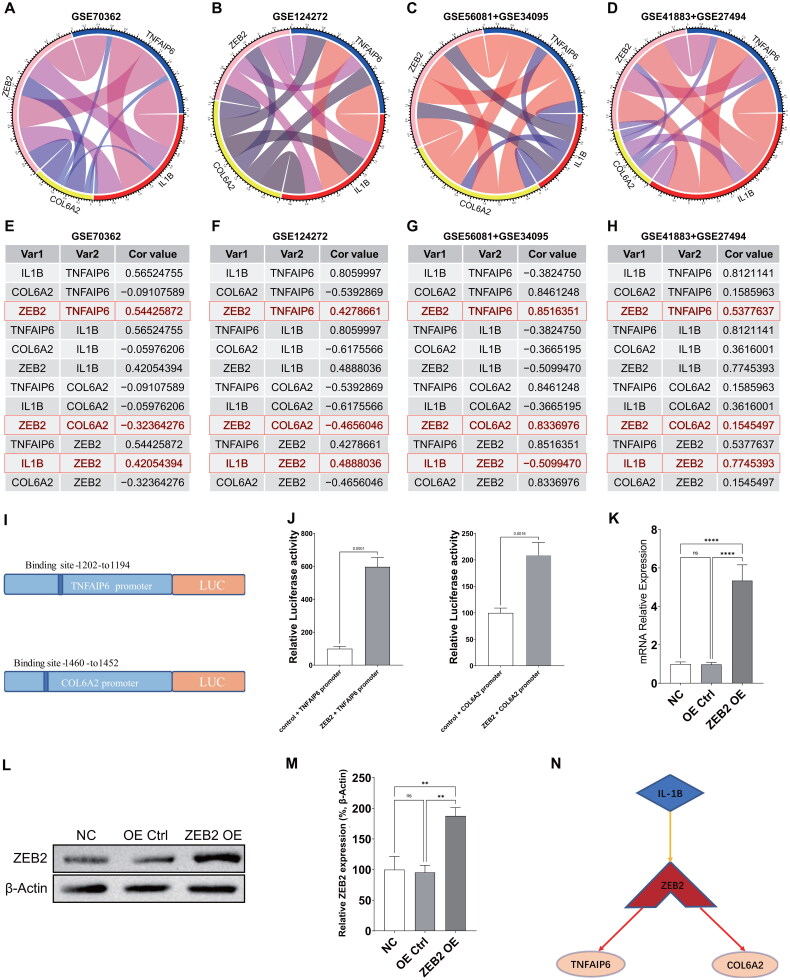
Correlation analysis was conducted among the TNFAIP6, COL6A2, IL-1β, and ZEB2 genes in four GEO datasets. (**A-D**) The correlation circle plots illustrate the relationships among these genes in the four GEO sets. (**E-H**) The grey images represent the correlation values of the four genes in the four GEO datasets. (**I**) The predicted promoter binding site of the two genes with ZEB2. (**J**) The luciferase reports provide confirmation of the interaction between the transcription factor (TF) and ZEB2 with the two hub genes. (**K**) The expression of ZEB2 targets was examined in both control and ZEB2-overexpressed 293 T cells using qPCR. (**L-M**) Western blot analysis was conducted to assess ZEB2 protein expression in ZEB2-overexpressed 293 T cells and controls. (**N**) The potential transcription factor regulatory network in IVDD was investigated. Data were represented as mean with SD. *p < 0.05, ***p < 0.001, **p < 0.01, ****p < 0.0001.

In order to examine the interaction network between transcription factors (TF) and the two hub genes, an analysis was conducted on the relationship between ZEB2 and TNFAIP6, COL6A2, or IL-1β using four publicly available datasets. The correlation circle plots (Figure 9 A-D) and corresponding correlation values (Figure 9 E-H) indicated that IL-1β showed a positive correlation with ZEB2 in three datasets, ZEB2 demonstrated a positive correlation with TNFAIP6 in all four datasets, and ZEB2 displayed a positive correlation with COL6A2 in only two datasets.

To further substantiate the interaction network between transcription factors (TF) and the two hub genes, we initially utilized JASPAR to predict the binding site of the two genes with ZEB2 (Figure 9 I). This was followed by plasmid transfection, construction testing, luciferase reporting, PCR, and WB testing (Figure 9 J-M). The luciferase reports provided evidence of the interaction between TF-ZEB2 and the two hub genes (Figure 9 J). Furthermore, in order to substantiate the regulatory effects of ZEB2, an investigation was conducted to assess the impact of ZEB2 overexpression on the expression profiles of 293 T cells. The results of qPCR (Figure 9 K) and WB (Figure 9 L-M) analysis confirmed a notable increase in ZEB2 expression in cells overexpressing ZEB2 compared to control cells. These findings suggest that the transcription factor ZEB2 interacts with two central genes, potentially leading to the upregulation of IL1B and subsequent regulation of the two key genes (Figure 9N). In summary, ZEB2 may serve as a crucial transcription factor involved in the regulation of key genes (TNFAIP6 and COL6A2) in the pathological processes of IVDD.

### 3.10. TNFAIP6 activated the TNFA_singaling_*via*_NFKB signaling pathway in IVDD

**Figure 10.**
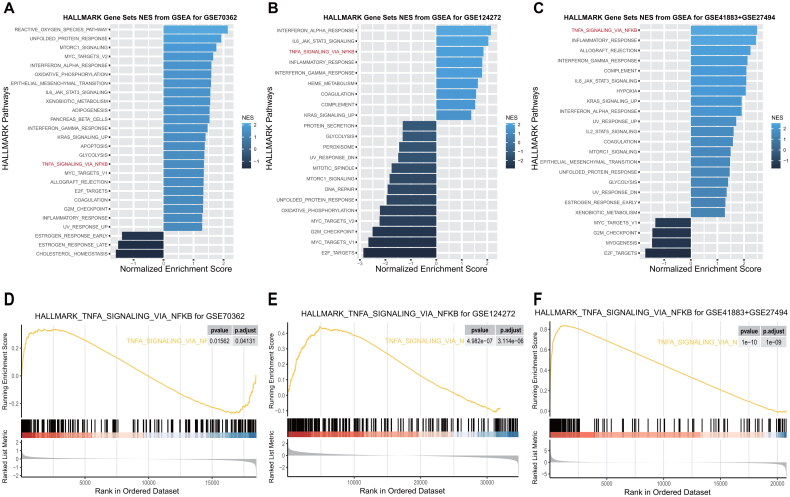
TNFAIP6 Activated TNFA_singaling_*via*_NFKB signaling pathway in IVDD. (**A-F**) GSEA analysis results of TNFAIP6 for three GEO datasets (GSE70362, GSE124272 and GSE41883 + GSE27494).

**Figure 11.**
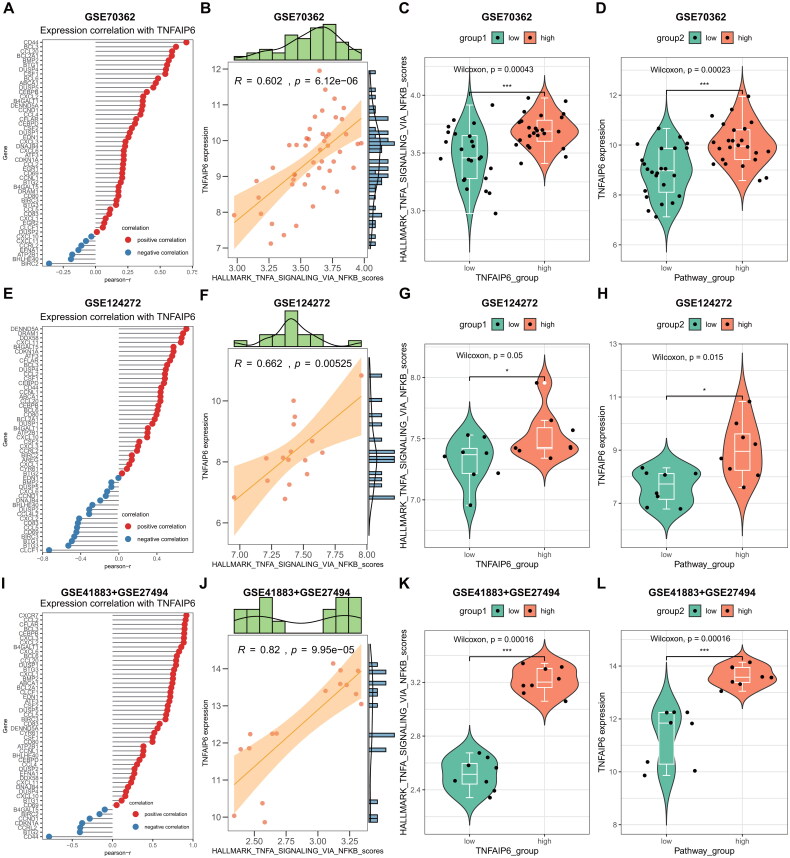
TNFAIP6 Correlated TNFA_singaling_*via*_NFKB signaling pathway in IVDD. (**A**) Correlation between TNFAIP6 and TNFA_singaling_*via*_NFKB signaling pathway genes in GSE70362 dataset. (**B**) Correlation between TNFAIP6 and TNFA_singaling_*via*_NFKB signaling pathway scores in GSE70362 dataset. (**C**) Comparison of the TNFA_singaling_*via*_NFKB signaling pathway scores between high and low TNFAIP6 expression subgroups in the GSE70362 dataset. (**D**) Comparison of TNFAIP6 expression between high and low subgroups of TNFA_singaling_*via*_NFKB signaling pathway scores in the GSE70362 dataset. (**E**) Correlation between TNFAIP6 and TNFA_singaling_*via*_NFKB signaling pathway genes in GSE124272 dataset. (**F**) Correlation between TNFAIP6 and TNFA_singaling_*via*_NFKB signaling pathway scores in GSE124272 dataset. (**G**) Comparison of the TNFA_singaling_*via*_NFKB signaling pathway scores between high and low TNFAIP6 expression subgroups in the GSE124272 dataset. (**H**) Comparison of TNFAIP6 expression between high and low subgroups of TNFA_singaling_*via*_NFKB signaling pathway scores in the GSE124272 dataset. (**I**) Correlation between TNFAIP6 and TNFA_singaling_*via*_NFKB signaling pathway genes in GSE41883 + GSE27494 dataset. (**J**) Correlation between TNFAIP6 and TNFA_singaling_*via*_NFKB signaling pathway scores in GSE41883 + GSE27494 dataset. (**K**) Comparison of the TNFA_singaling_*via*_NFKB signaling pathway scores between high and low TNFAIP6 expression subgroups in the GSE41883 + GSE27494 dataset. (**L**) Comparison of TNFAIP6 expression between high and low subgroups of TNFA_singaling_*via*_NFKB signaling pathway scores in GSE41883 + GSE27494 dataset.

The activation of the TNFA signaling pathway *via* NFKB was observed in all subclusters within the single-cell RNA sequencing data (Figure 10 A-F). Subsequently, an investigation into the specific mechanisms of TNFAIP6 in IVDD was conducted using GEO datasets. GSEA was performed on three GEO datasets (GSE70362, GSE124272, and GSE41883 + GSE27494) to identify altered signaling pathways between IVDD and control samples. Consistently, the TNFA signaling pathway *via* NFKB was found to be activated in all three datasets (Figure 10 A-F), underscoring its significance in IVDD pathogenesis. Additionally, a correlation analysis was conducted to examine the relationship between TNFAIP6 and the TNFA signaling *via* NFKB pathway using three GEO datasets. Encouragingly, TNFAIP6 exhibited a positive correlation with the majority of pathway genes, with CD44, DENND5A, CD80, CEBPB, CEBPD, and DUSP1/4 showing particularly high correlations with TNFAIP6 across all three datasets (Figure 11A, E, and I).

Moreover, the TNFA signaling *via* NFKB pathway was evaluated using the ssGSEA algorithm across three GEO datasets. The analysis revealed a positive correlation between TNFAIP6 and the TNFA signaling *via* NFKB pathway (Figure 11B, F and J). Distinct pathway scores were observed between high and low TNFAIP6 expression subgroups (Figure 11C, G and K), as well as between high and low pathway score subgroups (Figure 11D, H and L). These findings suggest that increased TNFAIP6 levels contribute to the progression of IVDD through activation of the TNFA signaling *via* NFKB pathway.

### 3.11. Experimental validation in IVDD tissues

**Figure 12.**
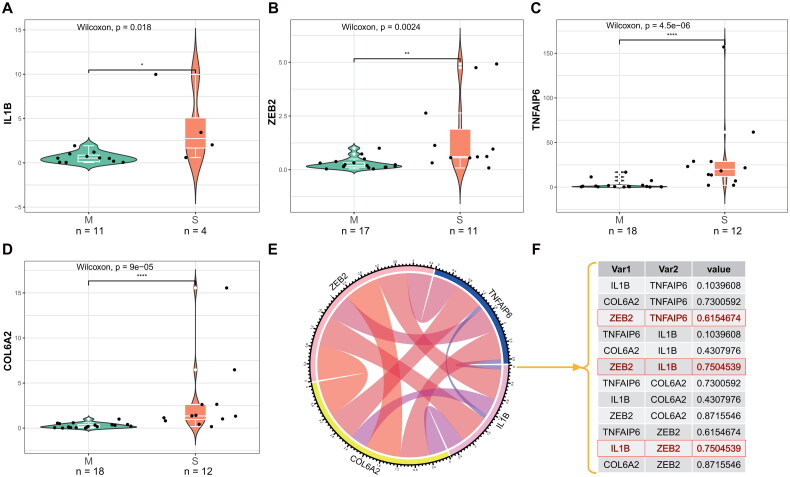
Verification of four genes by RT-qPCR. (**A-D**) The four genes (IL-1β (**A**), ZEB2 (**B**), TNFAIP6 (**C**) and COL6A2 (**D**)) showed significant upregulation in IVDD-severe tissue samples compared to IVDD-minor tissue samples. (**E**) Correlation circle pipes show the correlations among TNFAIP6, COL6A2, IL-1β and ZEB2 genes in ourself IVDD tissues. (**F**) The grey image depicts the Cor value of the four genes in in ourself IVDD tissues. AOD = Average optical density. *p < 0.05; **p < 0.01; ***p < 0.001.

In order to validate the potential transcription factor regulatory network, we conducted an analysis of the expression levels of IL-1β, ZEB2, TNFAIP6, and COL6A2 in intervertebral disc degeneration (IVDD) patients. Our findings demonstrate concordant changes between IVDD-M and IVDD-S tissue, suggesting a potential significant role in the IVDD process. Through RT-qPCR analysis of IVDD tissue samples, we observed significantly higher expression levels of the four genes in IVDD-S tissues compared to IVDD-M tissues (Figure 12 A-D). Additionally, correlation circle plots (Figure 12E) and correlation values (Figure 12F) revealed positive correlations among the four genes in our IVDD tissues.

**Figure 13.**
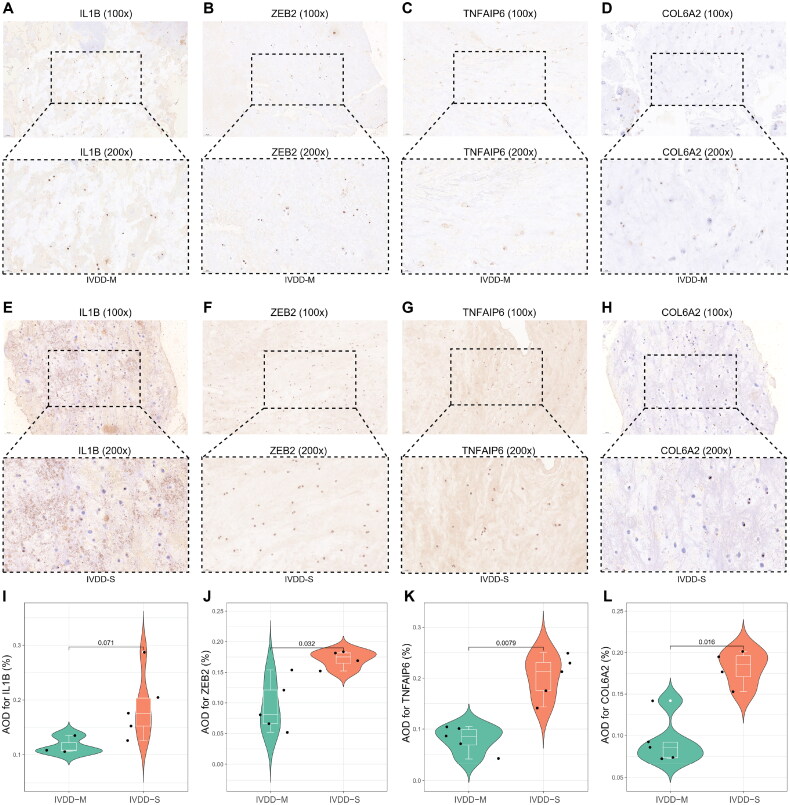
Verification of four genes for IHC. (**A-D**) IHC for IL-1β, ZEB2, TNFAIP6 and COL6A2 in the IVDD-M tissues. (**E-H**) IHC for IL-1β, ZEB2, TNFAIP6 and COL6A2 in the IVDD-S tissues. (**I-L**) The violin plots exhibit higher levels of staining for IL-1β, ZEB2, TNFAIP6, and COL6A2 protein expression in IVDD-S tissues as compared to IVDD-M tissues. AOD = Average optical density. *p < 0.05; **p < 0.01; ***p < 0.001.

To evaluate the internal index in intervertebral disc degeneration (IVDD) tissues, immunohistochemical staining was conducted on tissue sections to assess the expression of four proteins (Figure 13A-H). The findings indicated that IVDD-S tissues demonstrated elevated levels of staining for IL-1β, ZEB2, TNFAIP6, and COL6A2 protein expression in comparison to IVDD-M tissues (Figure 13I-L). These findings suggest that TNFAIP6 and COL6A2 may serve as useful diagnostic biomarkers for IVDD, while ZEB2 may function as a transcription factor in regulating TNFAIP6 during the pathological progression of IVDD

## 4. Discussion

Chronic low back pain has been linked to intervertebral disc degeneration (IVDD) and musculoskeletal disorders [38]. IVDD is comprised of an inner nucleus pulposus (NP) and an outer annulus fibrosus (AF), with the former containing a gelatinous extracellular matrix (ECM) rich in proteoglycans, primarily composed of aggrecan and type II collagen. This ECM is produced and maintained by a population of chondrocyte-like cells within the NP. The AF is primarily made up of concentric lamellae of type I collagen and cells with a fibroblast morphology and phenotype [38].

The pathogenesis of IVDD is multifaceted, with genetics, age, and lifestyle factors all playing a role. Inflammation is recognized as a crucial driver of IVDD progression, leading to matrix degradation, cellular senescence, and apoptosis. Inflammatory cytokines, particularly IL-1β and TNF-α, are key mediators of IVDD and associated low back pain, contributing to the onset and development of the inflammatory state and IVDD overall [16,39–42].

In this research, the hub genes TNFAIP6 and COL6A2 were identified through a combination of differentially expressed genes from GSE70362 and GSE124272 datasets, as well as single-cell RNA sequencing analysis. A generalized linear model (regression) was then constructed using these two genes, demonstrating a satisfactory diagnostic predictive value in both training and validation datasets. Subsequent validation through RT-qPCR (*p* < 0.05) confirmed the involvement of these genes and related inflammatory cytokines in tissues and whole blood samples. Next, an investigation was conducted to determine if ZEB2 acts as a potential key transcription factor in regulating TNFAIP6 and COL6A2 in intervertebral disc degeneration. Furthermore, GSEA, GSVA and ssGSEA analyses of the bulk-RNA data showed that the TNFAIP6 was correlated and activated with the TNFA_singaling_*via*_NFKB signaling pathway.

TNFAIP6, also known as TSG-6, is located in chromosome 2q23.3 [43]. The TNFAIP6 protein is a secreted member of the hyaluronic acid-binding protein family and plays a crucial role in ECM formation, inflammatory cell migration, cell proliferation and developmental processes [44,45]. It might contribute to the stability of the ECM and migration of the cells [46,47]. TSG‐6 is often combined with various ECM formation components, primarily stabilizing or reshaping ECM [47]. COL6A2 is located in zone 2 of the long arm of chromosome 21 (4), contributing to COL6 protein synthesis [48]. COL6A2 is a protein involved in the KEGG_ECM_RECEPTOR_INTERACTION signaling pathway (KEGG_ECM_RECEPTOR_ INTERACTION (gsea-msigdb.org)). Zhang et al. found that the COL6A2 gene promotes skeletal muscle cell proliferation, but its reduced expression reduces skeletal muscle cell proliferation, leading to skeletal muscle dysfunction and congenital muscle atrophy [49]. In the present study, TNFAIP6 was identified as the central gene associated with IVDD, exhibiting a significant upregulation as IVDD progressed. Conversely, COL6A2 displayed a negative correlation with IVDD progression. It is hypothesized that these two central genes are co-expressed and collaborate in promoting IVDD progression through extracellular matrix (ECM) interactions. This study not only enhances understanding of the mechanisms involved in IVDD development but also provides potential insights for therapeutic interventions.

TNFAIP6 is typically not expressed, but its expression is increased in various cells when exposed to inflammatory mediators and growth factors [50,51], particularly IL-1β and TNF-α. TNFAIP6, a type of TNF-α induced protein 2, has been shown to be upregulated in single-cell RNA sequencing data, four Gene Expression Omnibus (GEO) datasets, as well as our own tissue and whole blood samples. Similarly, IL-1β was found to be upregulated in single-cell RNA sequencing data, three GEO datasets, and our own tissue samples. These findings suggest that inflammatory mediators play a significant role in the development of intervertebral disc degeneration (IVDD). In addition, TNFAIP6 is also an inflammatory mediator in several autoimmune diseases and cancers [52,53], atherosclerosis [54], acute pancreatitis [55], type‐1 diabetes [56], osteoarthritis [53], acute lung injury [57] and glioblastoma [58].

It was confirmed that ZEB2 may serve as a potential key transcription factor regulating the expression of two key genes involved in the pathological process of IVDD. In the context of IVD degeneration, the upregulation of inflammatory mediator IL1B was observed to promote the expression of ZEB2. Additionally, our analysis of IVDD tissue samples revealed upregulation of TNFAIP6, ZEB2, and IL1B. It was hypothesized that the increased expression of ZEB2 led to the activation of TNFAIP6 and the inhibition of COL6A2, resulting in the upregulation of TNFAIP6 and the downregulation of COL6A2. To confirm the inference that TNFAIP6 and COL6A2 are involved in IVDD, additional basic cellular and animal experiments and sequencing data are required.

Furthermore, previous research has examined TNFAIP6 and COL6A2 in the context of IVDD [9]. Analysis has indicated that the six genes (CHI3L1, KRT19, COL6A2, DPT, TNFAIP6, and COL11A2) exhibited consistent alterations at both the protein and mRNA levels through comprehensive transcriptome and proteome assessments. While investigators evaluated the impact of these six genes on IVDD at the transcriptome and proteome levels, the study sample size was limited and lacked external validation. As such, their findings represent an initial exploration and warrant further confirmation with a larger cohort. TNFAIP6 and COL6A2 were screened in this study using integrated analysis of bulk-RNA seq and scRNA-seq datasets, yielding satisfactory results in both training and validation datasets. Subsequent RT-qPCR analysis of IVDD-related tissues and whole blood samples corroborated these findings, consistent with previous research [9]. Specifically, elevated levels of TNFAIP6 were observed in both tissue and serum samples from IVDD patients compared to healthy controls, suggesting TNFAIP6 and COL6A2 as a potential biomarker for assessing disease activity in IVDD.

GSEA was conducted to examine the gene set features of the high TNFAIP6 expression group. The results revealed a significant association between TNFAIP6 expression and the TNFA signaling *via* NFKB pathway in both bulk-RNA sequencing and single-cell RNA sequencing datasets. Furthermore, this correlation was validated across four IVDD levels using three GEO datasets. (1) Correlation between TNFAIP6 and TNFA_singaling_*via*_NFKB signaling pathway genes. (2) Correlation between TNFAIP6 and TNFA_singaling_*via*_NFKB signaling pathway scores. (3) Comparison of the TNFA_singaling_*via*_NFKB signaling pathway scores between high and low TNFAIP6 expression subgroups and (4) comparison of TNFAIP6 expression between high and low subgroups of TNFA_singaling_*via*_NFKB signaling pathway scores.

This study is subject to several limitations. Firstly, the sample size utilized in this investigation was relatively small, necessitating further studies with larger, independent samples to confirm the findings. Secondly, the focus of our study was on genes identified as differentially expressed in datasets, neglecting the analysis of certain characteristics such as sex and age in conjunction with the severity of IDD. Lastly, additional experiments are required to comprehensively elucidate the function of hub genes and explore the potential mechanisms underlying IVDD. Finally, to avoid analysis bias led by the retrospective nature of the current study, a prospective study should be performed in the future.

The current investigation has successfully identified and confirmed an upregulation in TNFAIP6 mRNA expression in individuals with intervertebral disc degeneration (IVDD). Our findings suggest that TNFAIP6 and COL6A2 may serve as potential biomarkers for predicting the diagnosis and progression of IVDD. Furthermore, TNFAIP6 has been shown to activate the TNFA signaling *via* NF-κB pathway, exacerbating the pathogenesis of IVDD. Further research involving larger sample sizes is warranted to elucidate the optimal clinical utility of TNFAIP6.

## Supplementary Material

Supplementary tables.xlsx

## Data Availability

The authors declare that all relevant data supporting the findings presented in this study are available within the article and its Supplementary Information files, or, from the corresponding author upon reasonable request.
